# Comparing the clinical outcomes of single vs. systematic dual stenting strategies for unprotected left main bifurcation lesion: a systematic review and meta-analysis

**DOI:** 10.3389/fcvm.2023.1145412

**Published:** 2023-07-24

**Authors:** Shuai Meng, Xiangyun Kong, Jing Nan, Xingsheng Yang, Jianan Li, Shenghua Yang, Lihan Zhao, Zening Jin

**Affiliations:** ^1^ Department of Cardiology and Macrovascular Disease, Beijing Tiantan Hospital, Capital Medical University, Beijing, China; ^2^ Department of General Medicine, Beijing Luhe Hospital, Capital Medical University, Beijing, China

**Keywords:** left main bifurcation, single stenting strategy, systematic dual stenting strategy, outcome, true left main bifurcation, complex PCI strategy for LM bifurcation

## Abstract

**Introduction:**

The optimal percutaneous coronary intervention (PCI) strategy for coronary left main (LM) bifurcation lesions remains controversial. This meta-analysis compared the medium and long-term follow-up clinical outcomes of single vs. systematic dual stenting strategies of LM bifurcation lesions.

**Methods:**

We systematically identified studies published within 5 years comparing single vs. systematic double stenting strategies for LM bifurcation lesions. The primary endpoint was medium-term (1 year) and long-term (at least 3 years) all-cause death. Secondary outcomes included major adverse cardiovascular events (MACEs), target lesion revascularization (TLR), overall occurrence of stent thrombosis (ST), cardiovascular (CV) mortality, and myocardial infarction (MI).

**Results:**

Two randomized controlled trials and nine observational studies with 7,318 patients were included in this meta-analysis. In terms of the medium-term follow-up clinical outcomes, our pooled analysis showed that use of the systematic dual stenting strategy was associated with a lower ST risk (odds ratio [OR] = 0.43, 95% confidence interval [CI]: 0.20–0.89, *P* = 0.02) and cardiac death risk (OR = 0.43, 95% CI: 0.21–0.89, *P* = 0.02) compared to the single stenting strategy; there was no significant difference between the two strategies regarding rates of all-cause death, MACE, TLR, and MI. Patients with long-term follow-up showed comparable observed clinical outcomes between the two strategies. Most importantly, for patients with true LM bifurcation, the risk of all-cause death, ST, and CV mortality following the systematic dual stenting strategy was significantly lower than the single stenting strategy.

**Conclusions:**

For patients with LM bifurcation lesions, both the systematic dual stenting strategy and single stenting strategy demonstrated comparable results in terms of all-cause mortality during medium-term and long-term follow-up. However, the systematic dual stenting strategy showed a tendency towards lower incidence of ST and CV mortality compared to the single stenting strategy during medium-term follow-up. Consequently, the systematic dual stenting strategy yielded superior clinical outcomes for patients with LM bifurcation lesions.

## Introduction

Despite great being advancements made in the field of devices, stenting techniques, and antithrombotic therapies, percutaneous coronary intervention (PCI) for coronary left main (LM) bifurcation lesions remains one of the most challenging procedures in real-world clinical practice. In addition, stenting for this subset of lesions is associated with suboptimal clinical outcomes in the early and long-term follow-up. LM bifurcation disease is detected in 5%–7% of patients undergoing coronary angiography, of whom 80%–90% suffer from distal LM bifurcation (1, 2). LM bifurcation lesions are usually characterized by a wider bifurcation angle, a greater area of the myocardium at risk of ischemia, and more frequent occurrence of a trifurcation lesion (3). These anatomical characteristics makes PCI for LM bifurcation more complex and challenging. However, the optimal PCI strategy for LM distal bifurcation disease remains controversial in current clinical practice. Therefore, we performed this systematic review and meta-analysis to determine whether the medium- and long-term clinical outcomes differ following the use of single or systematic dual stenting strategies for unprotected LM bifurcation lesions over the last 5 years. Furthermore, we assessed whether such differences are influenced by the location and complexity of the LM bifurcation lesion.

## Materials and methods

This systematic review and meta-analysis was performed following the recommendations of the Cochrane Handbook for Systematic Reviews of Interventions and Preferred Reporting Items for Systematic Reviews and Meta-Analysis statement (PRISMA). The PRISMA checklist is provided in Supplementary Table S1.

### Search strategy

A systematic literature search was performed using PubMed, Embase, and the Cochrane Library with the following terms: left main coronary artery bifurcation, LMCA bifurcation, left main bifurcation, and LM bifurcation; single stent, one stent, 1 stent, provisional; 2 stent, two stent, double stent, crush, culotte, DK-crush, mini-crush, T stenting, TAP, V stenting, Y stenting. The searching strategy for each database is provided in Supplementary Material. To identify all relevant studies, the reference from the eligible articles and reviews were also screened.

### Inclusion and exclusion criteria

Two authors (Shuai Meng and Xiangyun Kong) independently performed the study selection in concordance with the predefined PICOS (Participants, Intervention, Comparison, Outcomes, and Study design) criteria. Participants: patients with LM bifurcation lesion; Intervention: LM bifurcation lesion treated by systematic two-stent strategy; Comparison: LM bifurcation treated by the provisional/one-stent strategy; Outcomes: reporting medium-term (1 year) and long-term (>3 years) clinical outcomes of interest; Study design: randomized controlled trials (RCTs) or cohort studies published within 5 years (from January 2017 to November 2022).

Studies were further excluded based on the following criteria: (1) the important information could not be extract from the study; (2) study sample size of <100 patients; and (3) PCI for the LM bifurcation lesion was performed with bare metal stents.

### Data extraction and quality assessment

Extracted data included the following: study design, year of publication, follow-up data, baseline characteristics, cardiovascular (CV) comorbidities (dyslipidemia, hypertension, diabetes, previous history of PCI, chronic kidney disease, and cerebrovascular accidents), procedural characteristics (SYNTAX score, intravascular ultrasound utilization and trans-radial access rates), left ventricular ejection fraction, stent techniques used and clinical outcomes. For all binary outcomes, we extracted data regarding the number of events and the sample size of each group.

In addition, the risk of bias for the individual study was assessed by two investigators (Jing Nan and Xiangyun Kong), respectively. The revised Cochrane risk-of-bias tool for randomised trials (RoB 2) was used for randomised control trials (RCT) (4). For the non-randomized trials, the risk-of-bias tool for non-randomized studies for interventions (ROBINS-I) was implemented (5). The inter-rater agreement regarding the initial selection of included studies was 93%. Any disagreements or uncertainties with the final inclusion of 22 studies between the two reviewers in the processes of study selection, data extraction, and quality assessment were resolved by discussion with the senior investigator (Zening Jin).

### Definitions and outcomes

The systematic dual stent strategy included: crush, double kissing (DK)-crush, mini-crush, culotte, T stenting, T and small protrusion (TAP), and V stenting. Current-generation DES (C-DES) included second or third generation drug-eluting stents. Medium-term follow-up refers to a 1-year follow-up period; long-term follow-up indicates a minimum follow-up duration of 3 years. The primary endpoint was the medium-term (1 year) and long-term (at least 3 years) all-cause death. Secondary outcomes included major adverse cardiovascular vents (MACEs), target lesion revascularization (TLR), overall occurrence of stent thrombosis (ST), cardiovascular (CV) mortality and myocardial infarction (MI) at the medium-term and long-term follow up. LM bifurcation lesions were classified according to the Medina classification: 1,1,1 type, 1,0,1 type, and 0,1,1 type, which were defined as true bifurcation lesions (6).

### Statistical analysis

Continuous variables were presented as mean ± standard deviation (SD), while categorical variables were expressed as proportions. A comparison of the treatment with the systematic dual stenting strategy vs. the single strategy was performed used odds ratios (OR) and respective 95% confidence intervals (CI). The heterogeneity across the studies was assessed by Cochran's *Q*-test (*P* < 0.1 was regarded as statistically significant) and *I*^2^ statistics, which estimate heterogeneity quantitatively (*I*^2^-value <25% indicates no or mild heterogeneity, *I*^2^ > 75% indicates high heterogeneity). If *I*^2^ ≤ 50% and *P* ≥ 0.1, and the number of included studies of observed clinical outcomes was less than 5, then the fixed-effects model was used (7). If *I*^2^ > 50% or *P* < 0.1, data were pooled using random effects, according to the Mantel Haenszel model, and the cause of heterogeneity was sought. Publication bias assessment was made through visual inspection of the asymmetry in funnel plots. All statistical analyses were performed using Review Manager software (Rev-Man) version 5.3 (Cochrane Collaboration 2014, Nordic Cochrane Center, Copenhagen, Denmark). Two-tailed *P*-values <0.05 were considered significant.

## Results

### Study selection and quality

**Figure 1:**
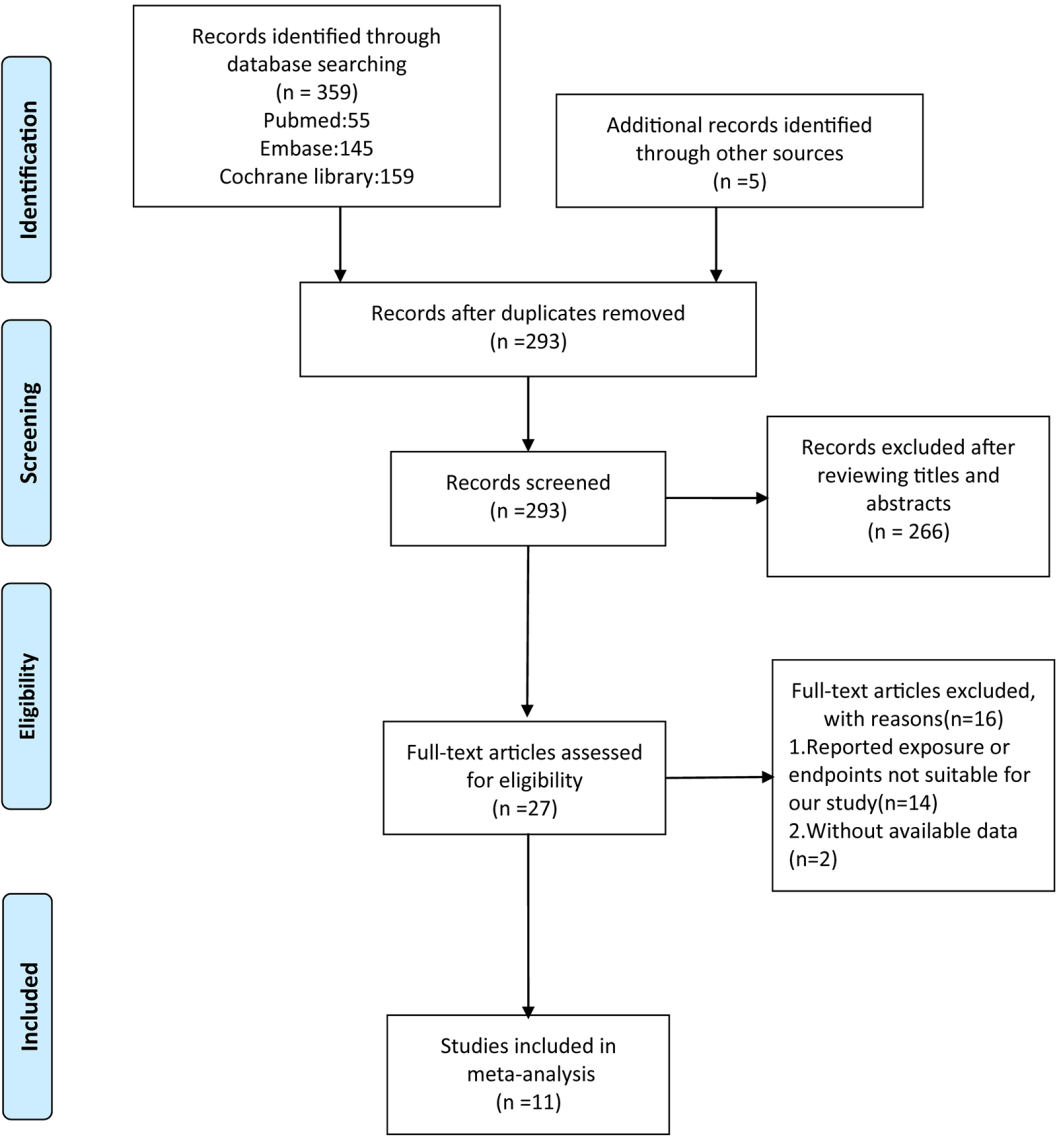
The flow chart of study selection.

In total, 359 articles were identified by a systematic search in PubMed (55), Embase (145), and the Cochrane library database (159), and five articles were identified from the reference lists of eligible studies. After removing duplicates and excluding articles not related to our topic, 27 articles underwent full-text assessment. According to the inclusion criteria, 11 studies were included in the present meta-analysis (8–18). The detailed flow diagram of study selection is presented in Figure 1. Of these, two were RCTs (8, 12) and nine were observational studies (9–11, 13–18, Table 1).

**Table 1:** General features of included studies.

Study	Registry	Study design	Country/Region	Study quality	Number of patients			Follow-up periods	Primary end point
					Total	Single stenting	Dual stenting		
Chen et al., 2019 (8, 19)	DKCRUSH-V	RCT	China, Indonesia, Thailand, USA, Ital	5	482	242	240	3 years	TLF (cardiac death, MI, TLR)
Cho et al., 2018 (9)	COBIS Ⅱ, KOMATE	Retrospective cohort study	Korea	8	464	385	79	3 years	MACE (cardiac death, MI, ST, TLR)
Choi et al., 2020 (10)	COBIS III	Retrospective cohort study	Korea	8	935	682	253	5 years	TLF (cardiac death, MI, TLR)
Ferenc et al., 2019 (11)	BBK-Left Main	Prospective cohort study	Germany (Single center)	8	867	477	390	10 years	MACE (all cause death, MI and TLR)
Hildick-Smith et al., 2021 (12)	EBC MAIN	RCT	Europe	5	467	230	237	1 year	Death, MI or TLR
Kandzari et al., 2018 (13)	EXCEL Trial	Prospective observational study	Canada, USA, Europe	8	529	344	185	3 years	MACE (cardiac death, MI, stroke, IDR)
Kawamoto et al., 2018 (14)	FAILS-2	Retrospective cohort study	Italy, Spain, Japan	7	377	216	161	3 years	MACE (all cause death, MI, TLR)
Lee et al., 2020 (15)	IRIS-DES, IRIS-MAIN	Prospective cohort study	Korea	8	1,002	440	562	3 years	TLF (cardiac death, TVMI, TLR)
Rhee et al., 2018 (16)	Grand-DES	Prospective cohort study	Korea	8	700	567	133	3 years	TLF (cardiac death, TVMI, TLR, all cause MI, ST)
Rigatelli et al., 2022 (17)	NA	Retrospective cohort study	Italy (Single center)	8	567	171	396	3 years	TLF (cardiovascular death, TVMI, TLR)
Wang et al., 2020 (18)	NA	Prospective cohort study	China (Single center)	8	928	444	484	3 years	MACE (cardiac death, MI or TVR)

RCT, randomized controlled trial; TLF, target lesion failure; MI, myocardial infarction; TLR, target lesion revascularization; MACE, major adverse cardiac events; ST, stent thrombosis; IDR, ischemic driven revascularization; TVMI, target vessel myocardial infarction; TVR, target vessel revascularization; NA, not available.

The primary endpoint, along with the definition of MACE for each trial, is presented in Table 1. Supplementary Table S2,S3 contain a summary of the risk of bias assessment. One of the RCTs was assessed as having some concerns in their overall risk of bias, mainly due to the deviation of assignment, in which trial five percent of patients allocated to the dual stent strategy had only a single stent implanted; twenty-two percent of patients in the stepwise provisional group had a second bifurcation stent implanted (12). As for the nine non-randomized clinical trials, the overall assessment for the risk of bias was serious for one study (15) and medium for the other four studies (8, 9, 16, 19), mainly derived from the confounding factors. Remaining four (11–14) were found to present low risk of bias.

### General characteristics of the included studies and patients

An overall assessment of the 11 eligible studies included data from 7,318 patients for inclusion in this meta-analysis. Of these, 4,198 received the single stenting strategy, whereas 3,120 received the systematic dual stenting strategy. Articles included were published from 2017 to 2022.

The general characteristics and CV risk factors of the 11 studies are summarized in Table 2. The baseline demographics and CV comorbidity rates of the enrolled patients who underwent the single or systematic dual stenting strategy were comparable (Table 2). As shown in Table 3, most patients used current-generation DES [In the study of Cho at al. (9) of the 1,353 patients included in the registry only those treated with C-DES were included in this meta-analysis], mostly with true LM bifurcation lesions. Furthermore, the systematic dual stenting strategy involved more than three bifurcation PCI technique in most studies.

**Table 2:** Baseline characteristics of included studies.

Author	Male *N* (%)	Age M ± SD	Hypertension *N* (%)	Dyslipidemia *N* (%)	Current smoker *N* (%)	Diabetes *N* (%)	Previous PCI *N* (%)	Previous MI *N* (%)	LVEF M ± SD	ACS (%)
	Single stenting/Systematic dual stenting strategy									
Chen	188 (77.7)/199 (82.9)	64 ± 10.0/65 ± 9.0	156 (64.5)/175 (72.9)	115 (47.5)/114 (47.5)	78 (32.2)/82 (34.2)	62 (25.6)/69 (28.8)	43 (17.8)/33 (13.8)	51 (21.1)/52 (21.7)	60 ± 9/59 ± 9	206 (85.1)/199 (82.9)
Cho	281 (73.0)/63 (79.7)	63.3 ± 10.3/65.9 ± 11.7	242 (62.9)/52 (65.8)	200 (51.9)/28 (35.4)	151 (39.2)/30 (38.0)	140 (36.4)/18 (22.8)	76 (19.7)/11 (13.9)	NA	56.3 ± 18.6/54.9 ± 18.4	170 (44.2)/47 (59.5)
Choi	524 (76.8)/187 (73.9)	65.0 ± 10.4/66.8 ± 11.3	419 (61.4)/138 (54.5)	280 (41.1)/81 (32.0)	172 (25.2)/54 (21.3)	262 (38.4)/94 (37.2)	114 (16.7)/45 (17.8)	35 (5.1)/13 (5.1)	58.5 ± 10.2/57.6 ± 10.3	405 (59.4)/153 (60.5)
Ferenc	357 (74.8)/291 (74.6)	70.6 ± 10.7/70.2 ± 10.9	404 (84.7)/326 (83.6)	NA	56 (11.7)/48 (12.3)	140 (29.4)/111 (28.5)	155 (32.5)/111 (28.5)	124 (26.0)/92 (23.6)	48.0 ± 9.7/49.0 ± 9.2	135 (28.3)/103 (26.4)
Hildick-Smith	182 (79.0)/177 (74.0)	70.8 (10.1)/71.4 (9.8)	180 (79.0)/190 (82.0)	158 (70.0)/166 (72.0)	36 (16.0)/30 (13.0)	66 (29.0)/62 (27.0)	93 (41.0)/99 (43.0)	60 (26.0)/66 (28.0)	NA	78 (33.9)/93 (39.2)
Kandzari	275 (79.9)/141 (76.2)	66.2 ± 9.3/66.8 ± 9.3	254 (73.8)/141 (76.2)	251 (73.0)/129 (70.1)	221 (64.8)/118 (64.1)	99 (28.8)/64 (34.6)	69 (20.1)/42 (22.8)	65 (19.2)/38 (20.8)	57.5 ± 9.4/56.1 ± 10.1	124 (50.6)/82 (44.3)
Kawamoto	170 (78.7)/128 (79.5)	70.8 ± 9.9/70.4 ± 10.4	181 (83.8)/126 (78.3)	150 (69.8)/104 (66.2)	24 (12.3)/25 (18.2)	98 (46.7)/59 (38.6)	114 (53.0)/79 (51.0)	80 (37.4)/43 (28.1)	55.0 ± 12.5/55.6 ± 9.9	NA
Lee	340 (77.3)/438 (77.9)	64.4 ± 10.5/64.4 ± 9.8	280 (63.6)/361 (64.2)	64 (14.5)/53 (9.4)	123 (28.0)/135 (24.0)	172 (39.1)/198 (35.2)	77 (17.5)/121 (21.5)	29 (6.6)/50 (8.9)	58.8 ± 10.5/59.6 ± 9.8	234 (53.2)/288 (51.2)
Rhee	426 (75.1)/94 (70.7)	65.2 ± 10.4/66.1 ± 11.1	340 (60.0)/77 (57.9)	374 (66.0)/90 (67.7)	137 (24.2)/30 (22.6)	214 (37.7)/54 (40.6)	NA	23 (4.1)/12 (9.0)	NA	336 (59.3)/82 (61.7)
Rigatelli	91 (53.2)/225 (56.8)	NA	NA	NA	NA	NA	NA	NA	NA	166 (97.1)/297 (75.0)
Wang	357 (80.4)/387 (80.8)	NA	251 (56.5)/285 (58.9)	245 (55.2)/263 (54.3)	148 (33.3)/165 (34.1)	117 (24.2)/149 (30.8)	127 (28.6)/145 (30.0)	148 (33.3)/142 (29.3)	NA	NA

M ± SD, mean ± standard deviation; PCI, percutaneous coronary intervention; MI, myocardial infarction; LVEF, left ventricular ejection fraction; NA, not available; ACS, acute coronary syndrome.

**Table 3:** Procedural characteristics of included studies.

Author	SYNTAX score M ± SD	Trans-radial access *N* (%)	IVUS utilization *N* (%)	True LM bifurcation (%)	C-DES (%)	Final balloon kissing dilatation *N* (%)	DAPT Duration	Systematic dual stenting technique (%)	Proportion of dual-stenting in the provisional arm
	Single stenting/Systematic dual stenting strategy								
Chen	30.1 ± 8.1/31.1 ± 7.9	181 (74.8)/187 (77.9)	98 (40.5)/103 (42.9)	100/100	100/100	191 (78.9)/239 (99.6)	Lifelong aspirin, clopidogrel 12 months	DK Crush	NA
Cho	23.0 ± 7.9/24.6 ± 7.9	NA	218 (56.6)/52 (65.8)	34.5/74.7	100/100	72 (18.7)/68 (86.1)	NA	Crush (45.6) T stent (30.4) Cullote (11.4) Kissing/V Stenting (3.8) Others7 (8.9)	NA
Choi	NA	366 (53.7)/106 (41.9)	427 (62.6)/172 (68.0)	20.7/75.5	96.9/95.2	163 (23.9)/233 (92.1)	NA	Crush (56.1%) T/TAP (23.7%) Culotte (6.3%) Kissing/V stenting (10.3)	NA
Ferenc	NA	NA	NA	42.3/83.3	71.1/65.4	NA	Lifelong aspirin, clopidogrel 6–12 months	TAP (88.2%) Culotte (10.8)	NA
Hildick-Smith	22.6 ± 5.9/23.2 ± 6.0	161 (71)/160 (70)	81 (36)/71 (31)	100/100	100/100	NA	NA	Culotte (51.1) T or TAP (32.1) Crush (DK) (4.6)	21%
Kandzari	27.8 ± 8.8/30.7 ± 8.7	135 (35.9)/38 (18.3)	266 (77.3)/139 (75.1)	34.3/77.0	100/100	189 (54.9)/156 (84.3)	3 years 61.8%)/3 years (57.2%)	T stent (50.8) Culotte (23.2) Crush/Mini-crush (14.4) V stent (6.1) Other (2.8)	NA
Kawamoto	29.0 ± 10.0/29.9 ± 10.0	24 (11.2)/21 (13.1)	48 (22.2)/44 (27.3)	100/100	100/100	NA	NA	Mini-crush (39.8) Culotte (32.9) T stent (14.3) Crush (7.5) Kissing/V Stenting (5.6)	9.7%
Lee	NA	NA	NA	100/100	77.5/72.2	NA	NA	NA	NA
Rhee	NA	NA	385 (67.9)/108 (81.2)	34.2/88.0	100/100	76 (13.4)/98 (73.7)	760.4 ± 391.6/773.0 ± 387.4	T/TAP (43.6) Crush (27.1) Culotte (19.5) Others (9.8)	NA
Rigatelli	NA			100/100	NA	396	12 months Ticagrelor or Prasugrel in ACS patients, 12-month Clopidogrel in the sand life-long aspirin	NIT (59.8) Culotte (24.7) TAP (15.4)	NA
Wang	NA	296 (66.7)/277 (57.2)	37 (8.3)/76 (15.7)	100/100	NA	263 (59.2)/465 (96.1)	Aspirin indefinitely, clopidogrel for at least 1 year	Mini-crush (56.4) Crush (51.2) DK crush (13.4) T stent (11.8) Kissing/V Stenting (9.5) Culotte (8.9)	NA

M ± SD, mean ± standard deviation; IVUS, intravascular ultrasound; LM, left main; C-DES, current-generation drug-eluting stent; DAPT, dual antiplatelet therapy; PCI, percutaneous coronary intervention; TAP, T and protrusion; NIT, nano-inverted-T; NA, not available.

### Medium-term clinical outcomes of the single vs. systematic dual stenting strategy

**Figure 2:**
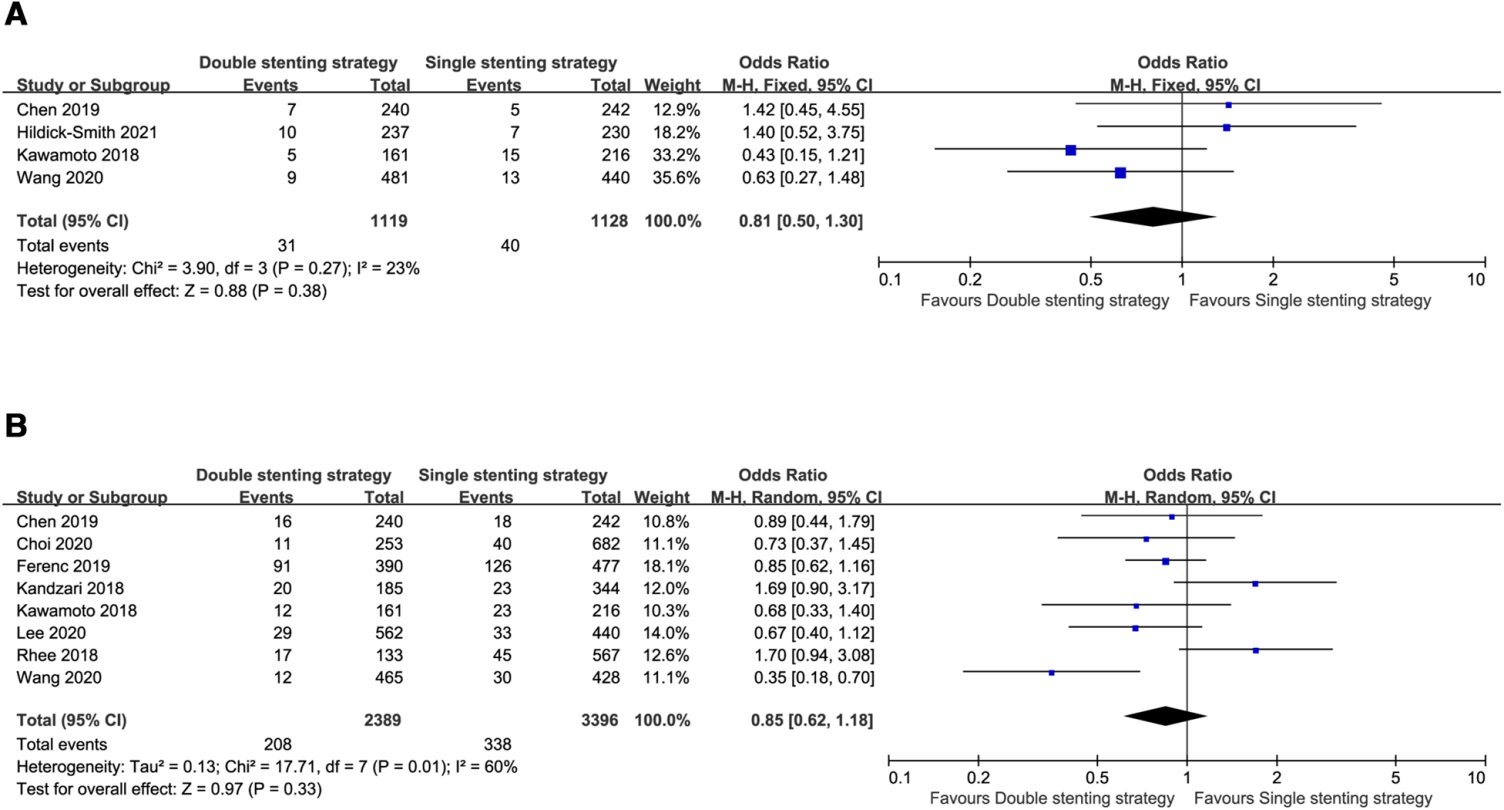
Forest plots of the primary outcome: single versus systematic dual stenting strategies for all-cause death at the medium-term and long-term follow-up. (**A**) All-cause death at the medium-term follow-up; (**B**) All-cause death at the long-term follow-up.

**Figure 3:**
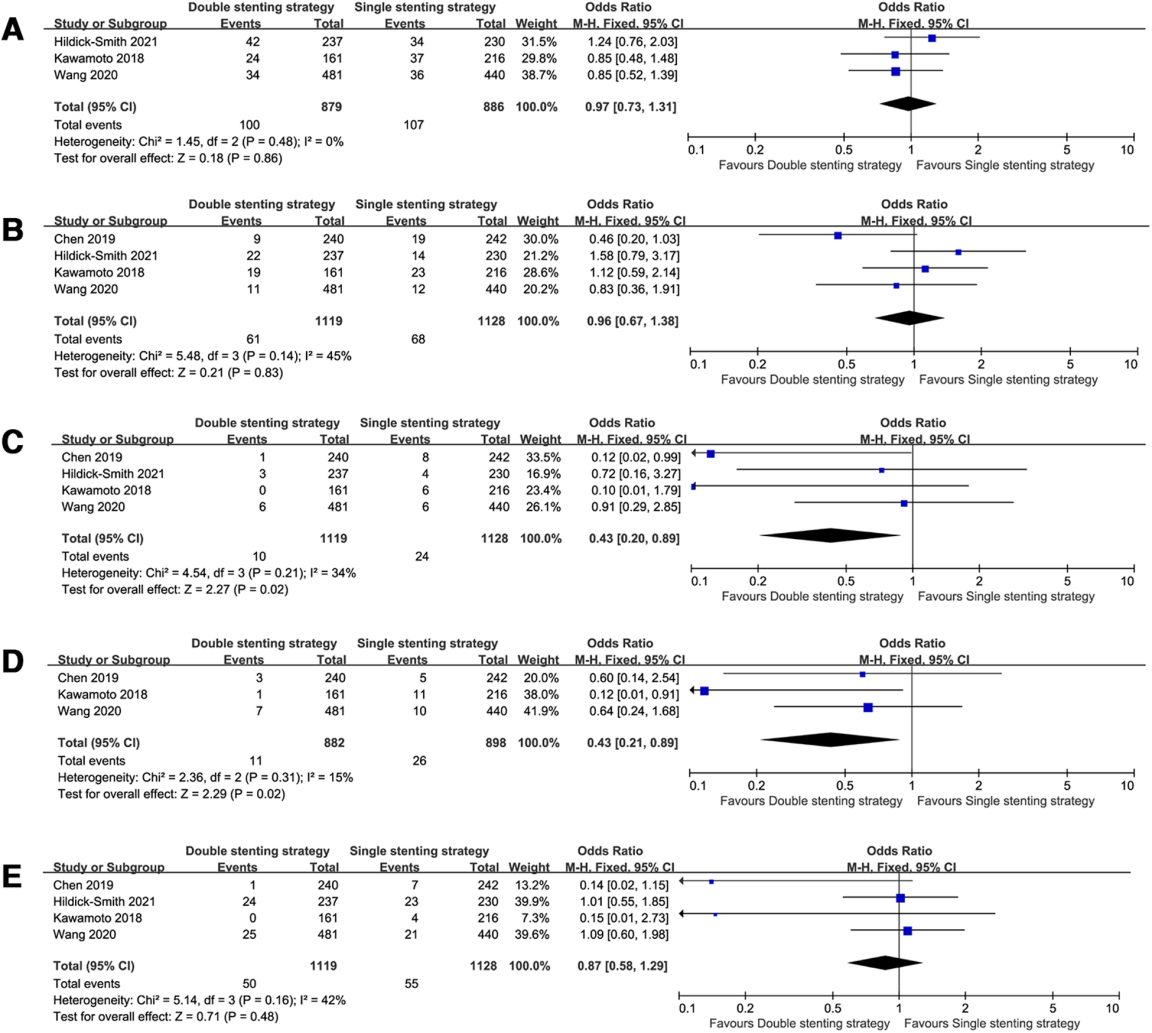
Forest plots of observed clinical outcomes at the medium-term follow-up. (**A**) Major adverse cardiac events (MACE); (**B**) target lesion revascularization (TLR); (**C**) stent thrombosis (ST); (**D**) cardiac death; (**E**) myocardial infarction (MI).

The risk of all-cause death (OR = 0.81, 95% CI: 0.50–1.30, *P* = 0.38, Figure 2) was comparable between the double stenting strategy and single stenting strategy at the medium-term follow-up. However, use of the double stenting strategy was associated with a lower risk of ST and CV mortality compared to the single stenting strategy (OR = 0.43, 95% CI: 0.20–0.89, *P* = 0.02; OR = 0.43, 95% CI: 0.21–0.89, *P* = 0.02, respectively, Figure 3). There were no significant differences between the two strategies in terms of MACE (OR = 0.97, 95% CI: 0.73–1.31, *P* = 0.86, Figure 3), TLR (OR = 0.96, 95% CI: 0.67–1.38, *P* = 0.83, Figure 3), or MI (OR = 0.87, 95% CI: 0.58–1.29, *P* = 0.48, Figure 3).

### Long-term clinical outcomes of the single vs. systematic dual stenting strategy

**Figure 4:**
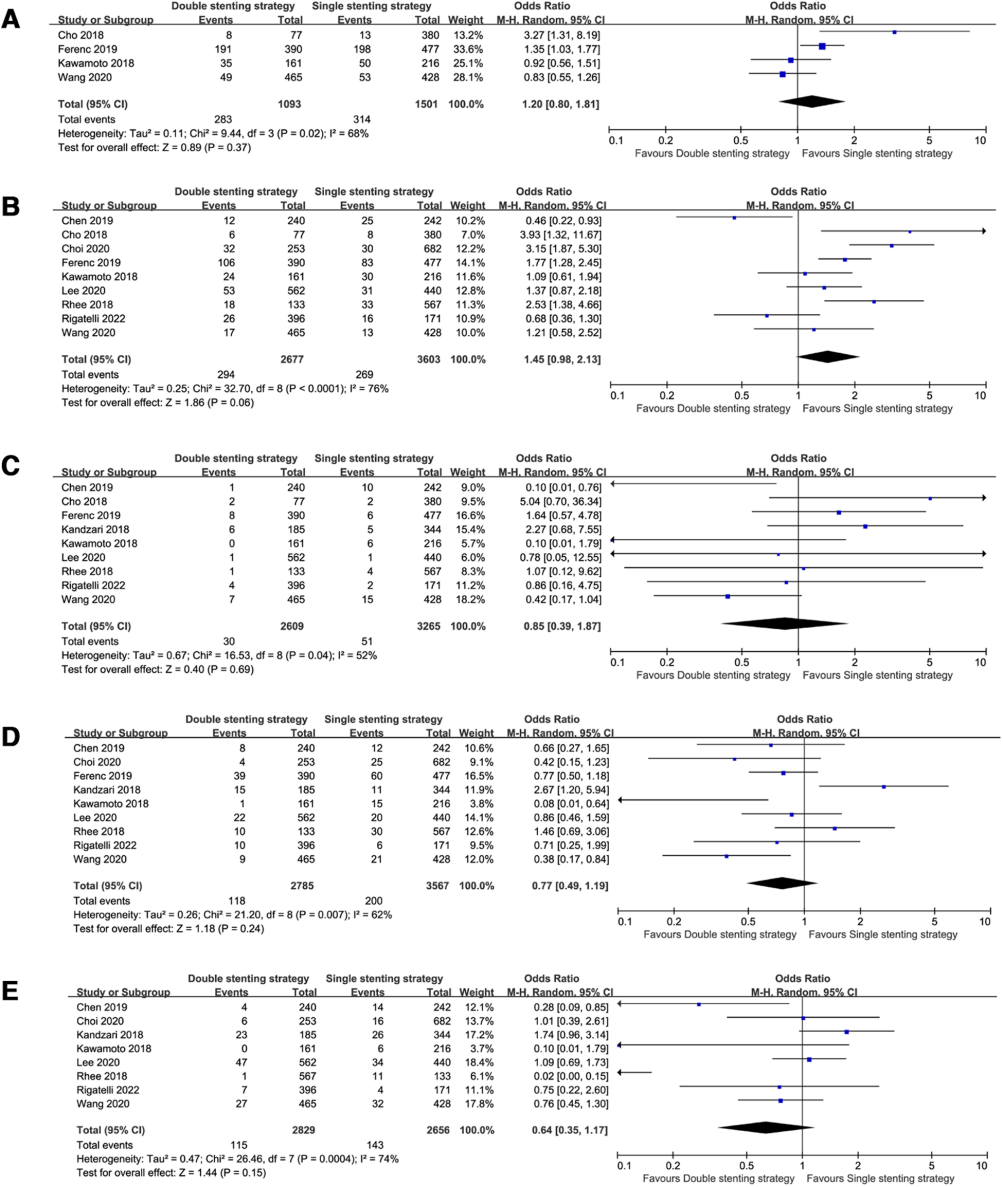
Forest plots of observed clinical outcomes at the long-term follow-up. (**A**) Major adverse cardiac events (MACE); (**B**) target lesion revascularization (TLR); (**C**) stent thrombosis (ST); (**D**) cardiac death; (**E**) myocardial infarction (MI).

When pooling the studies with >3 years follow-up data (8–11, 13–18), the observed clinical outcomes were comparable between the two strategies in the long-term follow-up, including all-cause death (OR = 0.85, 95% CI: 0.62–1.18, *P* = 0.33, Figure 2), MACE (OR = 1.20, 95% CI: 0.80–1.81, *P* = 0.37, Figure 4), TLR (OR = 1.45, 95% CI: 0.98–2.13, *P* = 0.06, Figure 4), ST (OR = 0.85, 95% CI: 0.39–1.87, *P* = 0.69, Figure 4), CV mortality (OR = 0.77, 95% CI: 0.49–1.19, *P* = 0.24, Figure 4), and MI (OR = 0.64, 95% CI: 0.35–1.17, *P* = 0.15, Figure 4).

### Single vs. systematic dual stenting strategies with special LM bifurcation

**Figure 5:**
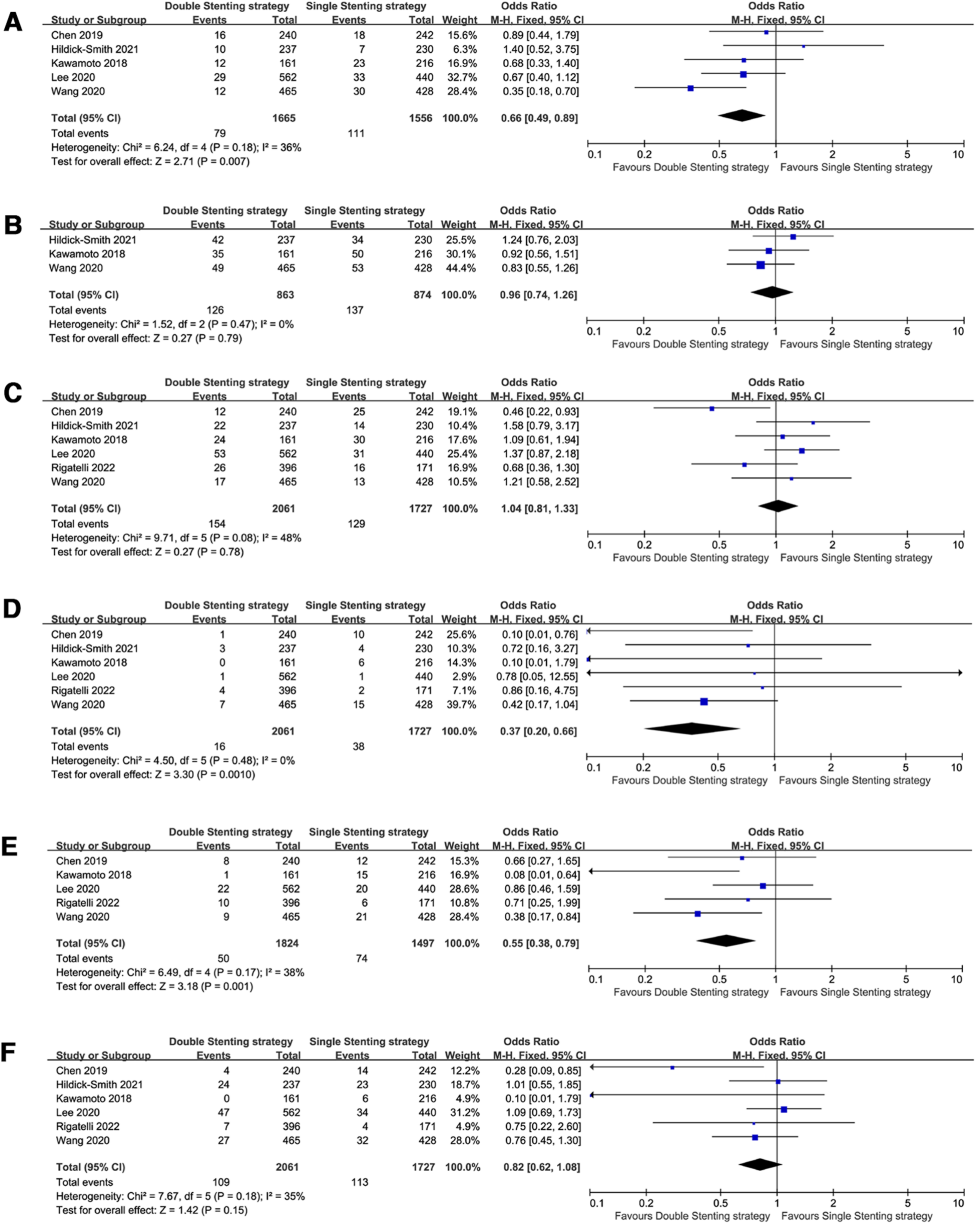
Forest plots of observed clinical outcomes with true left main (LM) bifurcation. (**A**) All-cause death; (**B**) Major adverse cardiac events (MACE); (**C**) target lesion revascularization (TLR); (**D**) stent thrombosis (ST); (**E**) cardiac death; (**F**) myocardial infarction (MI).

For true LM bifurcation trials (8, 12, 14, 15, 17, 18), only one trial among them showed the medium-term follow up (12), whereas others (8, 14, 15, 17, 18) included long-term follow-up data. Pooled analysis showed that the risk of all-cause death (OR = 0.66, 95% CI: 0.49–0.89, *P* = 0.007), ST (OR = 0.37, 95% CI: 0.20–0.66, *P* = 0.001), and CV mortality (OR = 0.55, 95% CI: 0.38–0.79, *P* = 0.001) in the double stenting strategy group were significantly lower than in the single stenting strategy group. The incidence rates were similar in terms of MACE, TLR, and MI, as demonstrated in Figure 5.

**Figure 6:**
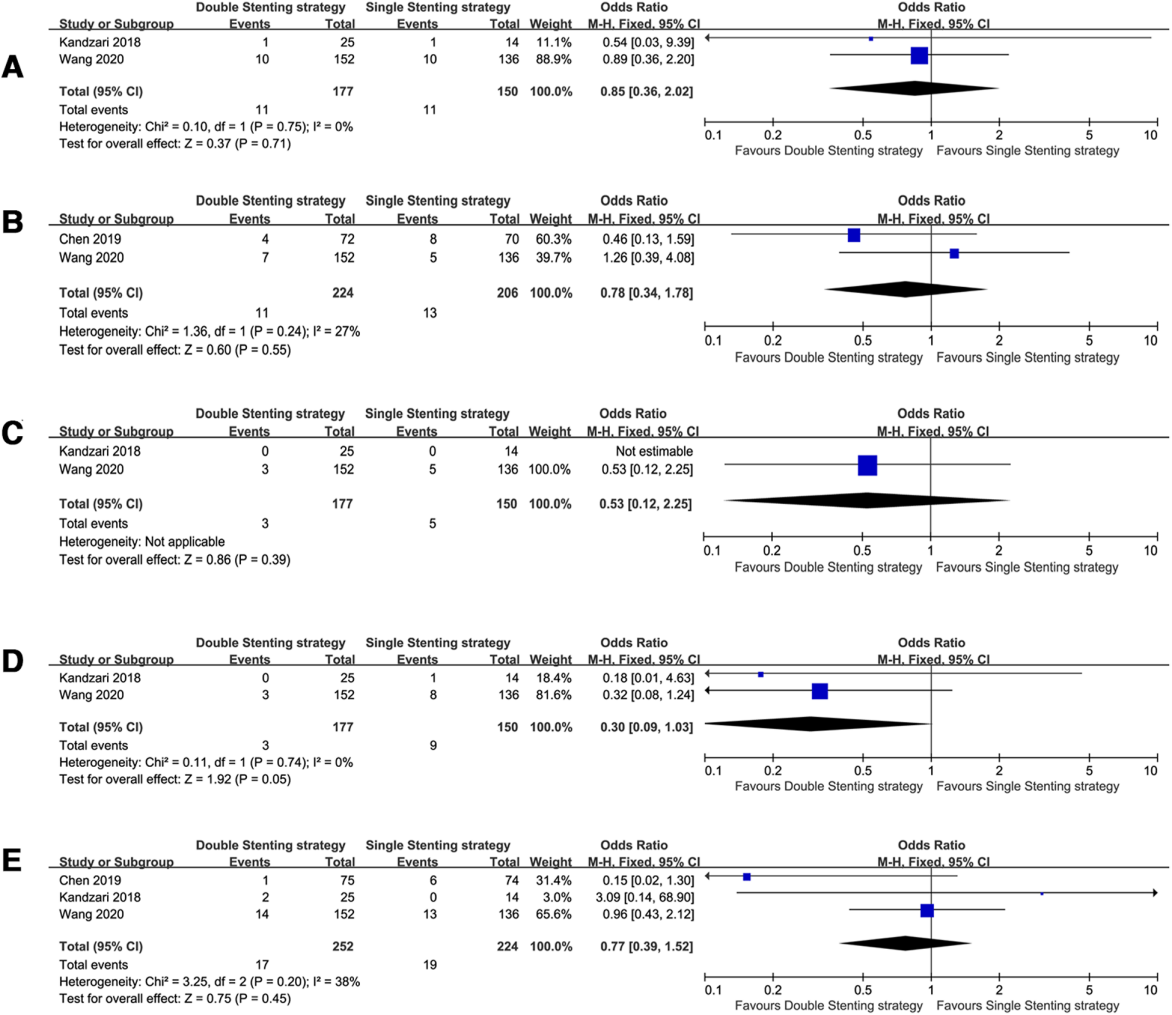
Forest plots of observed clinical outcomes with complex left main (LM) bifurcation. (**A**) All-cause death; (**B**) target lesion revascularization (TLR), (**C**) stent thrombosis (ST), (**D**) cardiac death, and (**E**) myocardial infarction (MI).

According to the DEFINITION criteria (20), three trials (8, 13, 18) with complex LM bifurcation and a long-term follow-up showed that CV mortality was decreased in patients who used the double-stenting strategy (OR = 0.30, 95% CI: 0.09–1.03, *P* = 0.05, Figure 6) compared with the single stenting strategy, albeit at a marginal significance level. However, all-cause death, TLR, ST, and MI rates were comparable between the two strategies.

### Publication bias and sensitivity analysis

Based on a visual inspection of the funnel plots (Supplementary Figures S1), there was no obvious publication bias for the TLR clinical outcomes using different stenting strategies; however, performance bias in these studies inevitably existed. The sensitivity analysis was performed by sequentially omitting one trial at a time, which also confirmed the main findings of our meta-analysis.

## Discussion

The main findings of the present systematic review and meta-analysis can be summarized as follows: (1) No statistically significant differences were noted between the systematic dual stenting strategy and single stenting strategy in terms of all-cause death; (2) the systematic dual stenting strategy was associated with a lower risk of ST and CV mortality compared to the single stenting strategy at the medium-term follow up; (3) the observed clinical outcomes were comparable between the two strategies at the long-term follow-up; and (4) for patients with true LM bifurcation, use of the systematic dual stenting strategy resulted in a lower rate of all-cause death, ST, and CV mortality than the single-stenting strategy.

We performed this meta-analysis of studies published in the last 5 years comparing the clinical outcomes with different bifurcation techniques. Overall, the results of our meta-analysis partly support the findings of the previous meta-analyses by Rigatelli et al., Abdelfattah et al., and Bhogal et al., that patients with LM bifurcation disease who used the double-stenting strategy had a similar all-cause death rate compared with the single-stenting strategy (21–23). Our results are in contrast to the meta-analysis including only RCTs with a follow-up ≥1 year that reported a lower rate of all-cause death with provisional stenting than with the two-stent strategy, although there were no differences observed in other endpoints, such as MI, TLR, and ST (24). However, the included trials were drawn between 2013 and 2017, while the wide application of modern stents and progress of stent technology, such as the DK-crush, and higher rates of intravascular imaging may contribute to better clinical benefits with the systematic double stenting strategy.

A previous meta-analysis suggested that the double-stent strategy was associated with a significantly higher risk of TLR, the differences were mainly driven by observational studies (22). Another meta-analysis drawn by Bhogal et al. suggested that provisional-stenting strategy was associated with a significant reduction of 3-year MACE compared with a dual-stenting strategy, primarily driven by TLR (23). We included more recent studies, including the European Bifurcation Club Left Main (EBC MAIN) trial, which found that the risk of TLR was comparable between the two strategies both at the medium and long-term follow-up. Another network meta-analysis of RCTs indicated that among various bifurcation techniques (crush, culotte, provisional or T stenting), the DK-crush technique was associated with fewer MACE and TLR events (25–27). Furthermore, this technique demonstrated superior outcomes in MI (26), as well as CV mortality and the incidence of ST (27).

Our pooled meta-analysis suggested that use of double stenting strategy was associated with a reduced risk of CV mortality and ST compared with the single stenting strategy at a medium-term follow up; however, these favorable clinical outcomes were no longer significant at the long-term follow-up between the two groups. These better clinical outcomes at the medium-term follow-up were mainly driven by the fact that the enrolled patients all had true LM bifurcation lesions. Furthermore, we also analysis trials studied with true LM bifurcation lesion, suggesting that the systematic dual stenting strategy was associated with reduced all-cause mortality, ST, and CV mortality. It is thought that the increase in the all-cause mortality risk of the single-stenting strategy most likely reflects increased CV mortality from ST. Therefore, the double-stenting strategy should be the first choice of operators in the management of true LM bifurcation lesions.

The European Society of Cardiology (ESC) and European Association for Cardio-Thoracic Surgery (EACTS) recommend the use of the DK-crush technique in true bifurcation lesions of the LM compared with the provisional technique; a class IIb recommendation was made for the DK-crush technique in complex LM bifurcations (28). On the other hand, the EBC still recommends the stepwise layered provisional stenting technique as the preferred strategy for most bifurcation lesions and distal LM lesions, not only when the use of a single stent is planned but also when the final use of two stents is anticipated before the procedure. The provisional stenting strategy is a philosophy that aims to keep the procedure as simple as possible and to reduce the number of needed stents in coronary bifurcation lesions (CBLs) (29–31). Yet, in more complex CBLs, especially those involving the LM lesion, adoption of dedicated two-stent techniques should be considered (32).

Improved stent technology and PCI techniques have made the management of complex LM bifurcation safer and more widely used (33, 34). Complex lesions are more likely to have characteristics (e.g., long ostial SB lesions) that prompt operators to use longer or multiple stents, which are associated with increased long-term events (35). The DK-crush strategy is recommended for complex bifurcations, with extensive side-branch disease and/or anticipated difficulty in re-accessing an important side branch (SB) (36). The Bifurcation Academic Research Consortium (Bif-ARC) proposes different criteria from angiography, intravascular imaging, as well as coronary CT aspects to define the complexity according to the method of evaluation (37).

Our subgroup analysis of complex LM bifurcation defined by the DEFINITION criteria (20) showed that the clinical outcomes were comparable between the systematic dual stenting and single stenting strategies. The DEFINITION II trial suggested that the systematic two-stent approach was associated with a significantly lower risk of MI as well as TLR compared with the provisional stenting approach in patients with complex coronary bifurcation lesions (38). Another study also suggested that, when faced with complex LM bifurcation disease, the double-stent strategy offers acceptable results in terms of CV death and ST, even in a challenging subset of patients, such as those with non-ST-elevation MI (39). It is imperative to standardize the definition of lesion complexity and trial design in future studies in the context of comparing complex bifurcation lesions (37).

Considering the pitfalls of the provisional stenting technique, the 16th EBC consensus document recommend a three-stage approach (ABC) to deployment of the first stent: stage A refers to the wiring of the main vessel (MV) and SB, stage B to MV and SB preparation, and stage C to stent implantation and optimization (40). This document provides a step-by-step overview of the pitfalls and technical troubleshooting during the implantation of the second stent in the provisional stenting strategy, when needed, and during stent implantation in upfront two-stent techniques (two-stent provisional stenting pathway and DK-crush stenting), when planned (41).

Some limitations of this meta-analysis should be acknowledged. First, observational studies have the inevitable presence of selection and ascertainment biases; the systematic dual stenting strategy tends to be used with complex lesion profiles and with minority patients (9, 16), leading to a relatively higher incidence of adverse events, which partially explains that the long-term benefits of the dual stenting strategy were not superior compared to the single stenting strategy. Second, although highly recommended for bifurcation treatment, imaging guidance techniques such as IVUS and optical coherence tomography (OCT) were not widely employed in the included trials. Furthermore, there was a significant discrepancy in the utilization of these imaging modalities among the studies included. Third, there is still no definitive consensus about the complexity of LM bifurcation lesions, we pooled our analysis of complex LM bifurcation lesions defined by the DEFINITION criterion, but was subject to the small simple size and lack of reporting; therefore, the results were underpowered to reveal the superiority of the dual stenting strategy. Moreover, most of the included studies used more than three dual-stent strategies, and the clinical outcomes for each strategy were not reported, precluding further clarification regarding the optimal choice of dual stent strategy. Besides, the slight differences in the end-point definitions, specific techniques, stent types, as well as operator expertise coupled with wide variability in the LM anatomy, might have contributed to the noted significant heterogeneity of the observed clinical outcomes in the long-term follow-up using the different techniques. Further RCTs of LM bifurcation disease still needed to provide more definite conclusions.

## Conclusions

In the present meta-analysis, the systematic dual stenting strategy was associated with a lower occurrence of ST and CV mortality compared to the single-stenting strategy at the medium-term follow up. The pooled data analysis suggested that regardless of the strategy used, the results were equivalent in terms of the long-term clinical outcomes. For true LM bifurcation lesions, the systematic dual stenting strategy showed better clinical outcomes.
